# Impact of Treatment Modalities on Discharge Disposition in Blunt Splenic Injuries

**DOI:** 10.7759/cureus.45987

**Published:** 2023-09-26

**Authors:** Kimberly Seal, Bryan Richmond, Sachin Jain, Jacob Minor, Tiffany M Lasky, Landon Reading, Damayanti Samanta

**Affiliations:** 1 Vascular Surgery, Charleston Area Medical Center, West Virginia University, Charleston, USA; 2 General Surgery, Charleston Area Medical Center, West Virginia University, Charleston, USA; 3 Critical Care, Charleston Area Medical Center, West Virginia University, Charleston, USA; 4 Trauma, West Virginia School of Osteopathic Medicine, Charleston, USA; 5 Trauma, Center for Health Services and Outcomes Research, Charleston Area Medical Center Institute for Academic Medicine, Charleston, USA

**Keywords:** independent risk factors, discharge disposition, treatment modality, blunt splenic injury, trauma

## Abstract

Background: Management of blunt splenic trauma has evolved over several decades, trending towards nonoperative management and splenic artery embolization. Extensive research has been conducted regarding the management of blunt splenic injuries, but there is little data on the association of treatment modality with discharge disposition.

Methods: This is an observational retrospective study conducted at a level-one trauma center with blunt splenic trauma patients of age ≥18 years between January 2010 and December 2021. The primary outcome of unfavorable discharge was defined as discharge to an acute care facility, intermediate care facility, long-term care facility, rehabilitation (inpatient) facility, or skilled nursing facility.

Results: Five hundred seventy-nine patients were included in the analysis, with 108 (18.7%) in the unfavorable group and 471 (81.3%) in the favorable group. Most patients were managed nonoperatively (69.3%), followed by splenectomy (25.0%) and embolization (5.7%). Due to the low number of embolizations performed during the study period, treatment modalities were grouped into two broad categories: intervention (embolization and splenectomies) and nonintervention. The treatment modality was found to have no significant impact on unfavorable discharge. Independent risk factors for unfavorable discharge included age >55 years, injury severity score (ISS) >15, hospital-acquired pneumonia, and in-hospital complications of sepsis.

Conclusions: This study provides an understanding of specific demographic and clinical factors that may predispose blunt splenic injury trauma patients to an unfavorable discharge. Providers may apply these data to identify at-risk patients and subsequently adapt the care they provide in an effort to prevent the development of in-hospital pneumonia and sepsis.

## Introduction

Blunt abdominal trauma is a common scenario encountered in the management of trauma patients. Among the abdominal organs that are injured in trauma, the spleen is the most common, with 23.8% of patients with abdominal trauma presenting with splenic injuries [1]. Management of blunt splenic injuries (BSI) has evolved recently, with recent literature suggesting that both minor (American Association for the Surgery of Trauma (AAST) grade I-II) and severe (AAST grade III-IV) can be effectively treated with nonoperative management (NOM) [2].

Splenic angiography and embolization are valuable adjuncts to nonoperative management. It was first described by Selafini in 1981 and has now become the treatment of choice in many trauma centers for the management of grade III or higher splenic injuries [3, 4]. The shift in paradigm toward NOM began in 1998 after Pachter et al. showed that 65% of all BSI could be managed nonoperatively, with a success rate of around 98% [5]. Since that time, there has been an abundance of literature published regarding the management of blunt splenic injuries, but there is little data on the impact that each treatment modality has on discharge disposition. This study aims to predict what factors and how each treatment modality impacts discharge disposition in patients with BSI.

## Materials and methods

This is a retrospective review of trauma patients admitted to Charleston Area Medical Center’s level-one trauma center located in Charleston, WV, USA. Patients included in this study were of age ≥18 years and experienced blunt splenic injuries with concomitant trauma between January 2010 and December 2021. Patients who had penetrating splenic injuries, had no imaging, left against medical advice (AMA), or were deceased prior to discharge were excluded from the study. Patients were grouped into two cohorts: those with favorable and unfavorable discharges. Favorable discharge was defined as discharge to home, whereas unfavorable discharge was discharge to an acute care facility, intermediate care facility, long-term care, rehabilitation (inpatient), or skilled nursing facility. Criteria for discharge to these facilities included any ongoing care that was required or any resultant disability that precluded safer discharge to home as determined by the attending physician. The aim of the study was to determine independent predictors of unfavorable discharge in blunt splenic trauma patients.

The study was reviewed and approved by Charleston Area Medical Center, West Virginia University Division's ethical board (approval number: 21-725). Following approval, patient data were obtained from the institutional trauma registry. Data points included patient demographics like age, gender, mechanism of injury (blunt or penetrating), injury severity score (ISS), pre-existing conditions, splenic injury grade, treatment modality, intensive care unit admission, in-hospital complications, and discharge disposition.

IBM SPSS software version 22.0 (IBM Corp., Armonk, NY, USA) was used for statistical analyses. Basic statistics of means and standard deviations for continuous variables and proportions and frequencies for categorical variables were conducted to describe study variables. Independent t-tests, or Mann-Whitney U, and chi-square, or Fisher’s exact, were used to compare patient demographics and characteristics between the two cohorts. In addition, logistic regression was conducted to identify the significant predictors of unfavorable discharge disposition. All probability values were two-sided, while p-values of <0.05 were considered significant. 

## Results

The study included 579 patients, with 108 (18.7%) in the unfavorable discharge group and 471 (81.3%) in the favorable discharge group. Patients with age ≥55 years (49.1% vs. 24.4%, p<0.001) and ISS >15 (87.0% vs. 58.2%, p<0.01) were significantly associated with unfavorable discharge. Moreover, patients with unfavorable discharge were more likely to have pre-existing cardiovascular disease (45.4% vs. 28.9%, p<0.01), diabetes (18.5% vs. 10.4%, p=0.01), autoimmune disease (3.7% vs. 1.1%, p=0.04) and renal disease (2.8% vs. 0.4%, p=0.01) (Table 1).

**Table 1:** Association of patient baseline characteristics with unfavorable discharge

	Unfavorable discharge (N=108)	Favorable discharge (N=471)	p-value
Age ≥ 55, n (%)	53 (49.1)	115 (24.4)	<0.001
Gender (male), n (%)	69 (63.9)	306 (65.0)	0.83
Injury severity score>15, n (%)	94 (87.0)	274 (58.2)	<0.001
Comorbidities, n (%)			
Alcohol/Substance use	47 (43.5)	277 (58.8)	<0.01
Neurologic	11 (10.2)	31 (6.6)	0.19
Hematologic	10 (9.3)	36 (7.6)	0.57
Pulmonary	2 (1.9)	8 (1.7)	0.91
Cirrhosis	4 (3.7)	11 (2.3)	0.41
Cardiovascular	49 (45.4)	136 (28.9)	<0.01
Diabetes	20 (18.5)	49 (10.4)	0.01
Psychiatric	15 (13.9)	48 (10.2)	0.26
Obesity	6 (5.6)	34 (7.2)	0.53
Autoimmune	4(3.7%)	5(1.1%)	0.04
Renal	3(2.8%)	2(0.4%)	0.01

With regard to the grade of splenic injury, the study found no significant association with the patient’s discharge disposition. The proportion of patients within each grade of splenic injury was comparable between the two groups. Of the treatment modalities, nonoperative management was the most common (69.3%), followed by splenectomy (25.0%) and embolization (5.7%). Due to the low number of embolizations performed during the study period, treatment modalities were grouped into two broad categories of intervention, including embolization and splenectomies, and the other category being nonintervention. Patients receiving intervention vs. nonoperative management had no significant impact on their discharge disposition (Table 2).

**Table 2:** Association of splenic injury grade and treatment modality with unfavorable discharge

	Unfavorable discharge (N=108)	Favorable discharge (N=471)	p-value
Splenic injury grade, n (%)			
Grade I	21 (20.0)	98 (21.4)	0.95
Grade II	26 (24.8)	102 (22.2)	
Grade III	31 (29.5)	147 (32.0)	
Grade IV	21 (20.0)	84 (18.3)	
Grade V	6 (5.7)	28 (6.1)	
Treatment modality, n (%)			
Intervention	40 (37.4)	137 (29.1)	0.09
No intervention	67 (62.6)	333 (70.9)	

However, ICU admission (96.3% vs. 86.0, p<0.01) and in-hospital complications of pneumonia (40.7% vs. 7.4%, p<0.01), sepsis (10.2% vs. 1.9%, p<0.01) and venous thromboembolism (pulmonary embolism/deep vein thrombosis (11.1% vs. 4.2%, p<0.01)) were found to be associated with unfavorable discharge (Table 3).

**Table 3:** Association of ICU incidence and in-hospital complications with unfavorable discharge

	Unfavorable discharge (N=108)	Favorable discharge (N=471)	p-value
Incidence of ICU, n (%)	104(96.3)	405(86.0)	<0.01
In-hospital complications, n (%)			
Venous thromboembolism (pulmonary embolism/deep vein thrombosis)	12 (11.1)	20 (4.2)	<0.01
Pneumonia	44 (40.7)	35 (7.4)	<0.001
Sepsis	11 (10.2)	9 (1.9)	<0.001

Finally, a logistic regression was conducted with all significant risk factors identified in the univariate analysis. In-hospital complication of pneumonia was the strongest predictor (odds ratio (OR) =6.15, 95% confidence interval (CI) 3.44-10.98, p<0.001), followed by ISS >15 (OR = 4.69, 95% CI 2.42-9.08, p<0.001), age ≥ 55 years (OR = 3.79, 95% CI 2.10-6.82, p<0.001) and in-hospital complication of sepsis (OR = 3.66, 95% CI 1.26-10.65, p=0.01) (Table 4).

**Table 4:** Logistic regression determining the independent risk factors for unfavorable discharge

	p-value	Odds ratio	95% Confidence interval	
			Lower	Upper
Age ≥ 55 years	<0.001	3.79	2.10	6.82
Injury severity score >15	<0.001	4.69	2.42	9.08
In-hospital complications of pneumonia	<0.001	6.15	3.44	10.98
In-hospital complications of sepsis	0.01	3.66	1.26	10.65

## Discussion

Discharging trauma patients from the hospital could be challenging due to their unique characteristics of high acuity and complexity, socioeconomic issues, and an unidentified payor source [6]. Delay due to post-hospital placement acts as the primary barrier to patient discharge. Postponement of patient discharge leads to increased hospital length of stay, which is associated with a higher direct cost of hospitalization. Discharge delays are also linked to emergency department crowding associated with adverse patient outcomes and experiences. Moreover, delays in discharge make coordination difficult among providers, consultants, social workers, and patients or their families [7].

Some studies in the trauma literature have focused on identifying independent predictors of discharge disposition. The goal of these studies was to identify demographic, clinical, and hospital factors in the overall trauma population that could help with planning discharge early during hospitalization [6-9]. A few studies, however, have concentrated on discharge disposition in specific conditions such as falls, traumatic brain injury, and traumatic thoracic aortic injury [10-12]. Discharge disposition in elderly trauma patients has also been well studied [13, 14]. There is no study to date examining predictors for discharge disposition in patients with blunt splenic trauma when blunt abdominal trauma is a common presentation and the spleen is the most commonly injured abdominal organ [1]. Discharge disposition in the existing literature has been categorized as home and non-home (nursing home, hospice, rehabilitation center, another hospital) [6, 7, 11]. The non-home category of these studies aligns with the unfavorable discharge disposition of the current study.

The current study examined the impact of patient demographics, injury severity, pre-existing conditions, splenic injury grade, treatment modality, and hospital factors on discharge disposition in patients with blunt splenic trauma. Around 19% of the patients in the study had an unfavorable discharge disposition, which is similar to the data presented in the existing trauma literature, with non-home discharge ranging from 23.1% to 36% [6, 7, 9, 11]. Likewise, the predictors of discharge disposition identified in the current study, including older age and injury severity, were comparable to those established by previous research. In a study conducted by Khorgami et al. with data from the Oklahoma State Trauma Registry, age ≥50 years and ISS ≥15, among other variables, were found to predict discharge to a facility. The likelihood of discharge to a facility was five-fold in patients with ages ≥ 50 years and 1.5-fold in patients with ISS ≥15 [6]. In a more recent study, Graham et al. developed and validated a predictive model for discharge disposition with data from the National Trauma Database. The study reported older age and higher ISS, in addition to other predictors, as independent risk factors for non-home discharge. The likelihood of non-home discharge was four times higher in patients aged ≥65 years and 80% less in patients with ISS ≤8. Similar impacts of age and ISS were established in the current study. The likelihood of unfavorable discharge was almost four-fold in patients aged ≥ 55 years and five-fold in patients with ISS > 15 [7].

Hospital-acquired pneumonia and in-hospital complications of sepsis were also found to predict unfavorable discharge in the current study. Patients with hospital-acquired pneumonia were six times more likely to have an unfavorable discharge, whereas patients developing sepsis in the hospital were four times more likely to have an unfavorable discharge. It is interesting to note that none of the existing studies have included in-hospital complications in their predictive models [6-10]. In-hospital complications can negatively impact patient recovery and disrupt their discharge to a residential environment, in addition to other adverse outcomes and an increased length of stay [15]. Providers should examine their treatment plans and care processes to identify areas of improvement that may help prevent complications.

Findings from this study provide insight concerning factors that predispose blunt splenic trauma patients to have an unfavorable discharge disposition. Application of these data may facilitate the identification of patients at risk for unfavorable discharge and serve to guide providers in managing these at-risk patients by taking appropriate measures to prevent the development of in-hospital pneumonia and sepsis. Despite the study adding novel data to the existing literature, it is not without limitations. The retrospective design of the current study makes it reliant on the identification, availability, and completeness of patients’ medical records. Accordingly, all patients admitted to the trauma center during the study period may not have been identified and included, resulting in selection bias. In addition, the validity of the evaluated data depends on the accuracy with which it has been documented and subsequently collected. Moreover, the primary objective of the study was to determine the impact of treatment modality on discharge disposition; however, the study included a limited sample size of embolization. Future studies should be conducted with an adequate and comparable representation of different treatment modalities to assess their impact on discharge disposition.

## Conclusions

The spleen is the most commonly injured organ in blunt abdominal trauma patients. The management of blunt splenic traumas has evolved over time, especially in favor of nonoperative management. It is crucial to identify independent risk factors, including treatment modality, that predispose blunt splenic injury trauma patients to an unfavorable discharge. These data will aid providers in identifying at-risk patients and adapting patient care to prevent the development of complications.
